# Cysteamine Attenuates the Decreases in TrkB Protein Levels and the Anxiety/Depression-Like Behaviors in Mice Induced by Corticosterone Treatment

**DOI:** 10.1371/journal.pone.0026153

**Published:** 2011-10-19

**Authors:** Ammar Kutiyanawalla, Alvin V. Terry, Anilkumar Pillai

**Affiliations:** 1 Department of Psychiatry and Health Behavior, Georgia Health Sciences University, Augusta, Georgia, United States of America; 2 Department of Pharmacology and Toxicology, Georgia Health Sciences University, Augusta, Georgia, United States of America; 3 Medical Research Service, Charlie Norwood Veterans Affairs Medical Center, Augusta, Georgia, United States of America; University of Queensland, Australia

## Abstract

**Objective:**

Stress and glucocorticoid hormones, which are released into the circulation following stressful experiences, have been shown to contribute significantly to the manifestation of anxiety-like behaviors observed in many neuropsychiatric disorders. Brain-derived neurotrophic factor (BDNF) signaling through its receptor TrkB plays an important role in stress-mediated changes in structural as well as functional neuroplasticity. Studies designed to elucidate the mechanisms whereby TrkB signaling is regulated in chronic stress might provide valuable information for the development of new therapeutic strategies for several stress-related psychiatric disorders.

**Materials and Methods:**

We examined the potential of cysteamine, a neuroprotective compound to attenuate anxiety and depression like behaviors in a mouse model of anxiety/depression induced by chronic corticosterone exposure.

**Results:**

Cysteamine administration (150 mg/kg/day, through drinking water) for 21 days significantly ameliorated chronic corticosterone-induced decreases in TrkB protein levels in frontal cortex and hippocampus. Furthermore, cysteamine treatment reversed the anxiety and depression like behavioral abnormalities induced by chronic corticosterone treatment. Finally, mice deficient in TrkB, showed a reduced response to cysteamine in behavioral tests, suggesting that TrkB signaling plays an important role in the antidepressant effects of cysteamine.

**Conclusions:**

The animal studies described here highlight the potential use of cysteamine as a novel therapeutic strategy for glucocorticoid-related symptoms of psychiatric disorders.

## Introduction

Chronic stress and elevated levels of glucocorticoids have been implicated in psychiatric disorders including anxiety, depression and schizophrenia [1]. In addition, chronic exposure to glucocorticoids has been shown to manifest anxiety like symptoms in rodents [2]. Brain derived neurotrophic factor (BDNF) signaling through its receptor TrkB plays an important role in neuroplasticity [3]–[5], and chronic stress has been known to result in alterations in BDNF/TrkB signaling and changes in neuronal functions [6]–[7]. Moreover, human postmortem studies have revealed changes in TrkB mRNA and protein levels in schizophrenia [8] and suicide subjects [9]. The argument that TrkB signaling plays an important role in anxiety and depression has been further strengthened by a recent study which indicated that mice lacking TrkB exhibit increased anxiety-like behaviors [10].

Many studies have shown that the prevalence of anxiety symptoms in schizophrenia and depression patients is higher than in the general population indicating the importance of developing new treatment strategies for this aspect of the disorders [11]–[12]. Cysteamine, the FDA-approved drug currently prescribed for cystinosis, has anti-oxidant properties [13]. Evidence also indicates that short-term treatment of mice with cysteamine increases brain as well as serum BDNF levels in rodents [14]–[15]. A recent study from our laboratory has shown that cysteamine increases TrkB signaling in mouse frontal cortex [15].

In the present study, we evaluated the potential of cysteamine to attenuate chronic corticosterone-induced changes in TrkB protein levels and anxiety/depression-like behaviors in mice. Furthermore, the possible role of TrkB in mediating cysteamine effects was examined using TrkB knockout mice.

## Results

Each mouse typically ingested approximately 6 ml of water per day (24 h) and gained approximately 10 g over the course of the study. However, no significant difference in relative body weight gain during the experiment or water intake was found between any of the treatment groups (data not shown)

### Chronic corticosterone treatment downregulates TrkB protein levels in mouse frontal cortex and hippocampus

The expression of the BDNF receptor, TrkB was examined in frontal cortex and hippocampus after 3, 5 and 7 weeks of corticosterone treatment. In the frontal cortex, two-way ANOVA revealed a significant main effect of treatment [F(1,25) = 6.063; p = 0.02] and a significant treatment x time interaction [F(2,25) = 3.304; p = 0.05], without a significant effect of time [F(2,25) = 1.639; p = 0.214]. Post hoc analysis indicated that treatment with corticosterone for 3 (Fig 2A) and 5 (Fig 2B) weeks did not change TrkB protein levels, but 7-week treatment resulted in a significant decrease in TrkB protein levels (Fig 2C; p<0.05). In hippocampus, there was a significant main effect of time [F(2,24) = 16.07; p<0.0001] and treatment [F(1,24) = 34.63; p<0.0001],without a significant treatment x time interaction [F(2,24) = 1.295; p = 0.292]. Interestingly, TrkB protein levels in the hippocampus were significantly decreased in groups treated with corticosterone for 3 (Fig 3A;p<0.01), 5 (Fig 3B; p<0.01) and 7 (Fig 3C; p<0.05) weeks. We also examined the protein levels of truncated TrkB in the above brain areas following corticosterone treatment; however, we did not find any change in truncated TrkB levels in any of the treatment groups (data not shown). To determine whether TrkB downregulation occurred at the mRNA level, we examined TrkB mRNA expression in the frontal cortex and hippocampus from the 7-week corticosterone treated mice. TrkB mRNA levels in corticosterone-treated mice did not differ from vehicle-treated mice (Fig 4).[Fig pone-0026153-g001]

**Figure 1 pone-0026153-g001:**
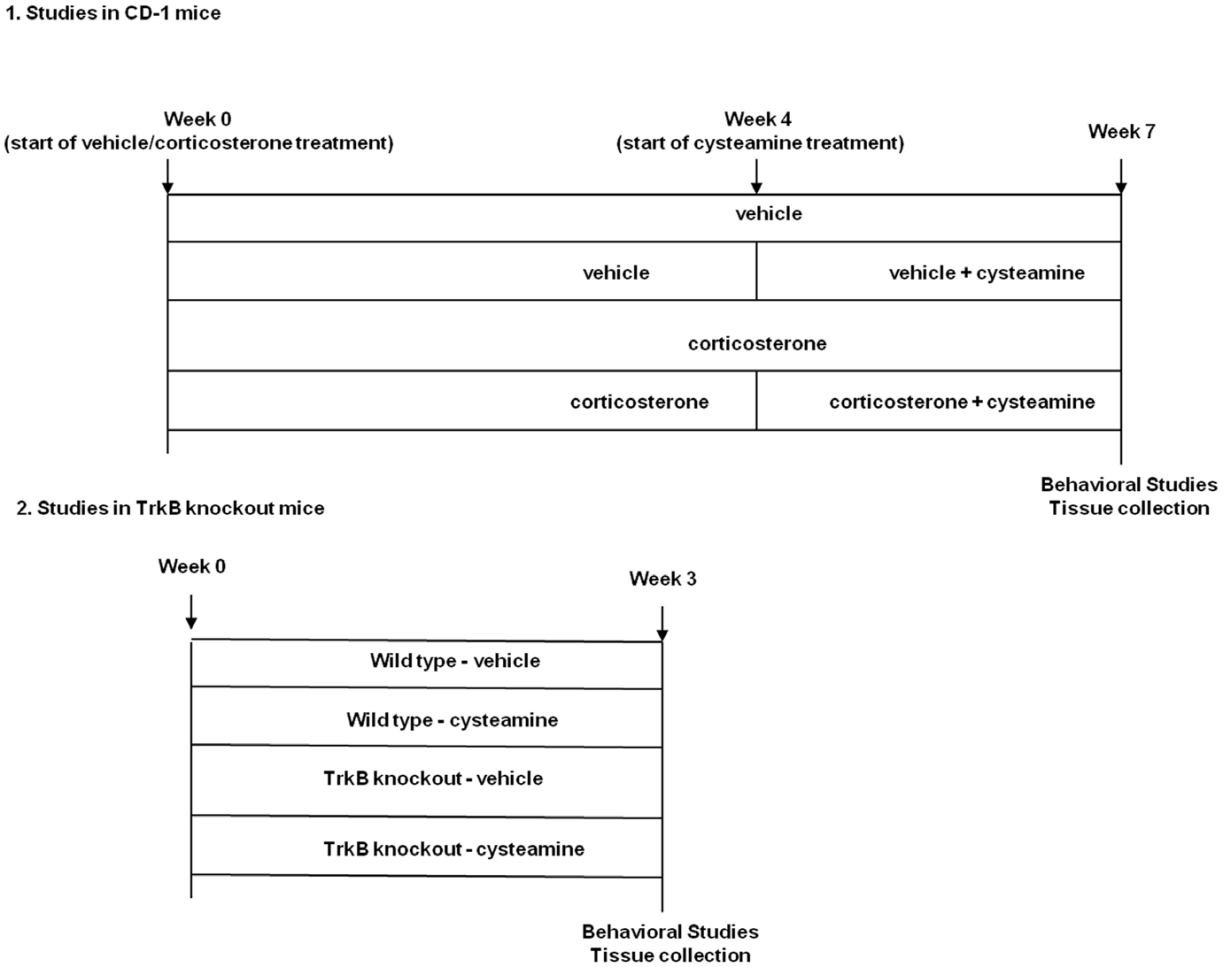
Experimental designs used for testing the behavioral and biochemical responses to cysteamine treatment in CD-1 and TrkB knockout mice. (1) CD-1 male mice were treated for 7 weeks with corticosterone (CORT; 35 ug/ml/day) or vehicle (0.45% hydroxypropyl-β-cyclodextrin) in presence or absence of cysteamine (150 mg/kg) during the last three weeks of corticosterone or vehicle treatment. At the end of the treatment, the same animal was successively tested in the Open Field (OF) paradigm, the Light/dark test, the Elevated Plus Maze test (EPM), the Tail Suspension Test (TST) and then sacrificed for protein or mRNA analysis. (2) Cysteamine (150 mg/kg) or water (vehicle) was administered through drinking water to TrkB knockout and wild type mice for 21 days. At the end of the treatment, mice were killed and brains removed for behavioral and biochemical analyses.

**Figure 2:**
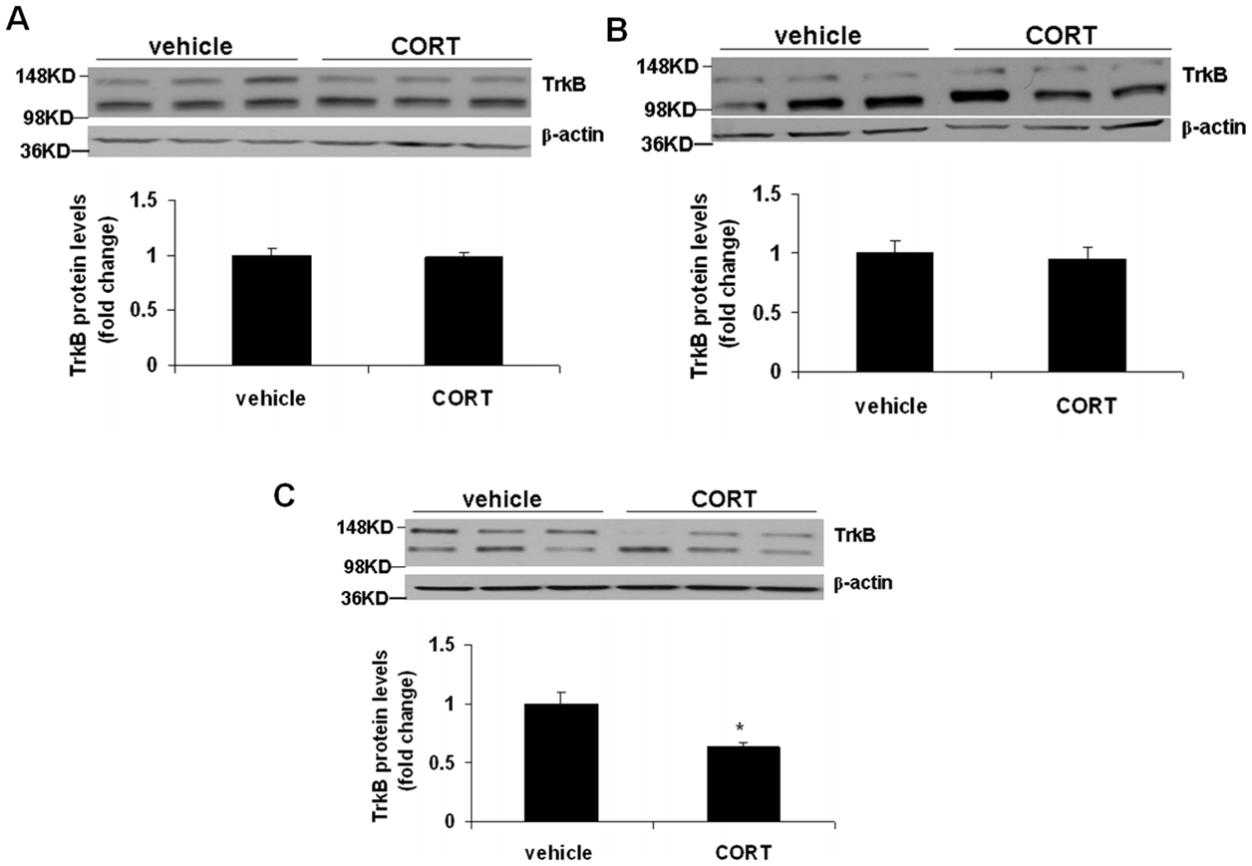
Effects of chronic corticosterone treatment on TrkB protein levels in the frontal cortex. CD-1 male mice were treated with corticosterone (CORT; 35 ug/ml/day) or vehicle (0.45% hydroxypropyl-β-cyclodextrin) for (A) 3, (B) 5 or (C) 7 weeks. TrkB protein levels were determined by Western blot analysis. The upper panel shows a representative autoradiogram of TrkB and the lower panel represents the fold change in optical density values normalized to vehicle-treated controls. β-actin was used as a protein loading control. Values are mean ± SE (n = 5–6 mice per group). *p<0.05 versus vehicle.

**Figure 3:**
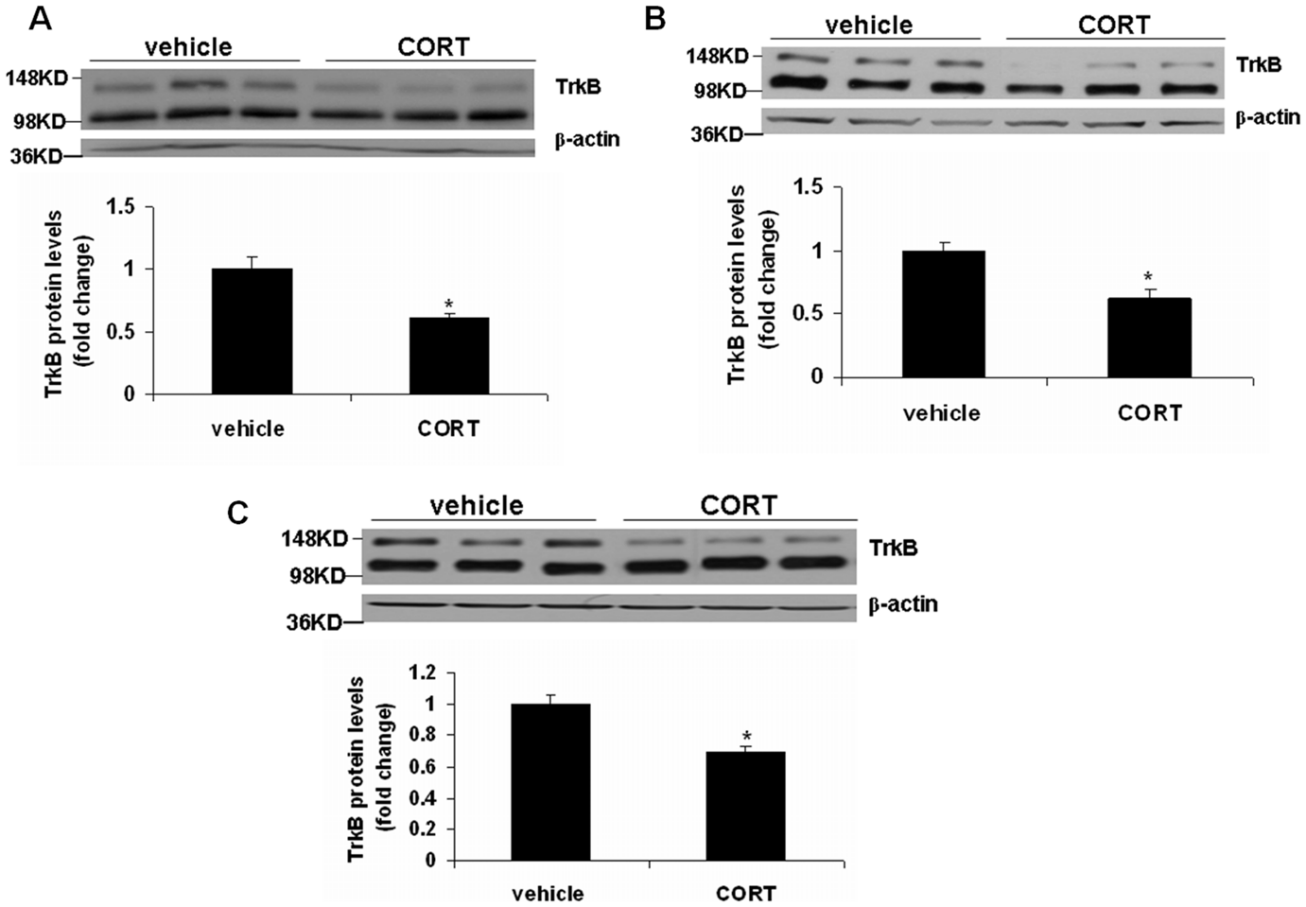
Effects of chronic corticosterone treatment on TrkB protein levels in the hippocampus. CD-1 male mice were treated with corticosterone (CORT; 35 ug/ml/day) or vehicle (0.45% hydroxypropyl-β-cyclodextrin) for (A) 3, (B) 5 or (C) 7 weeks. TrkB protein levels were determined by Western blot analysis. The upper panel shows a representative autoradiogram of TrkB and the lower panel represents fold change in optical density values normalized to vehicle-treated controls. β-actin was used as a protein loading control. Values are mean ± SE (n = 5–6 mice per group). *p<0.05 versus vehicle.

**Figure 4:**
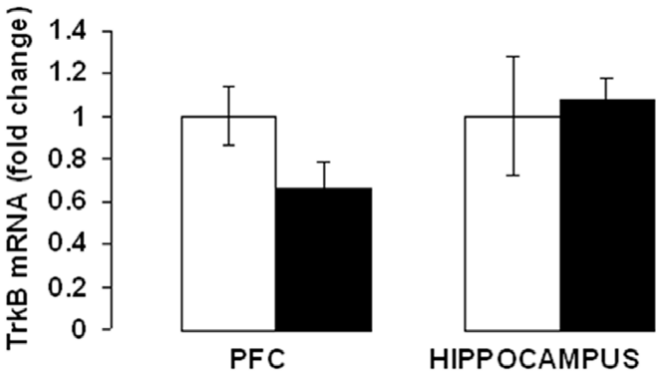
Effects of chronic corticosterone treatment on TrkB mRNA levels in the frontal cortex and hippocampus. CD-1 male mice were treated with corticosterone (CORT; 35 ug/ml/day) or vehicle (0.45% hydroxypropyl-β-cyclodextrin) for 7 weeks. TrkB mRNA levels were determined by qRT-PCR analysis. The level of TrkB mRNA was normalized to that of RPS3 RNA in the same sample. Values are expressed as fold change relative to vehicle-treated mice. Open and filled bars represent vehicle and corticosterone-treated groups, respectively. Error bars represent standard Error (SE) of n = 4 mice per group.

### Cysteamine attenuates chronic corticosterone-induced decrease in TrkB protein levels

**Figure 5:**
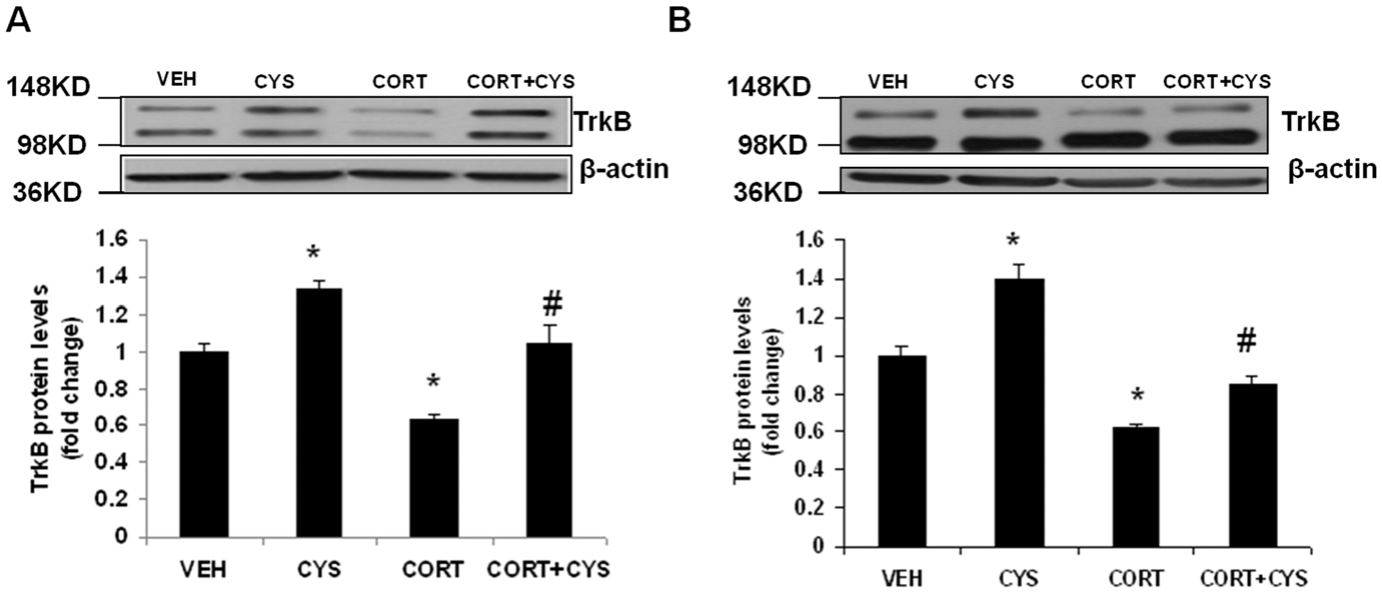
Effects of cysteamine in chronic corticosterone-treated mice on TrkB protein levels in the frontal cortex and hippocampus. CD-1 male mice were treated for 7 weeks with vehicle (0.45% hydroxypropyl-β-cyclodextrin) or corticosterone (CORT; 35 ug/ml) in the presence or absence of cysteamine (CYS; 150 mg/kg/day) during the last three weeks of corticosterone treatment. TrkB protein levels were determined in the (A) frontal cortex and (B) hippocampus by Western blot analysis. The upper panel shows a representative autoradiogram of TrkB and the lower panel represents the fold change in optical density values normalized to vehicle-treated controls. β-Actin was used as a protein loading control. Values are mean ± SE (n = 5–6 mice per group). *p<0.05 versus vehicle and ^#^p<0.05 versus CORT.

Subsequently, we determined if cysteamine could reverse corticosterone-induced decreases in TrkB protein levels. CD-1 mice were treated for 7 weeks with vehicle or corticosterone in the presence or absence of cysteamine during the last three weeks of corticosterone treatment. In the frontal cortex, two-way ANOVA with corticosterone/vehicle pretreatment and cysteamine/vehicle as main effects indicated significant main effect of pretreatment [(F(1,18) = 34.69; p<0.0001] and treatment [F(1,18) = 26.81; p<0.0001], but without a significant pretreatment x treatment interaction [F(1,18) = 0.3669; p = 0.552]. Post hoc analysis with Bonferroni's multiple comparison test showed that treatment with cysteamine significantly increased TrkB protein levels in frontal cortex (Fig 5A). The increase in TrkB protein levels was observed in both corticosterone (p<0.001) and non-corticosterone treated animals (p<0.05). Similar to frontal cortex, data from hippocampus showed significant main effect of pretreatment [F(1,18) = 40.84; P<0.0001] and treatment [F(1,18) = 88.46; p<0.0001]. No significant effect was found in pretreatment x treatment interaction in hippocampus [F(1,18) = 2.729; p = 0.118]. The reduction in TrkB protein levels in the hippocampus induced by chronic corticosterone was reversed by cysteamine (Fig 5B; p<0.05). We did not find any significant change in truncated TrkB levels following cysteamine treatment in the vehicle or corticosterone treated groups (data not shown). Taken together, these results suggest that chronic cysteamine treatment is effective in reversing the reductions in TrkB protein levels induced by excess glucocorticoids.

### Cysteamine treatment did not change BDNF and plasma corticosterone levels

**Figure 6:**
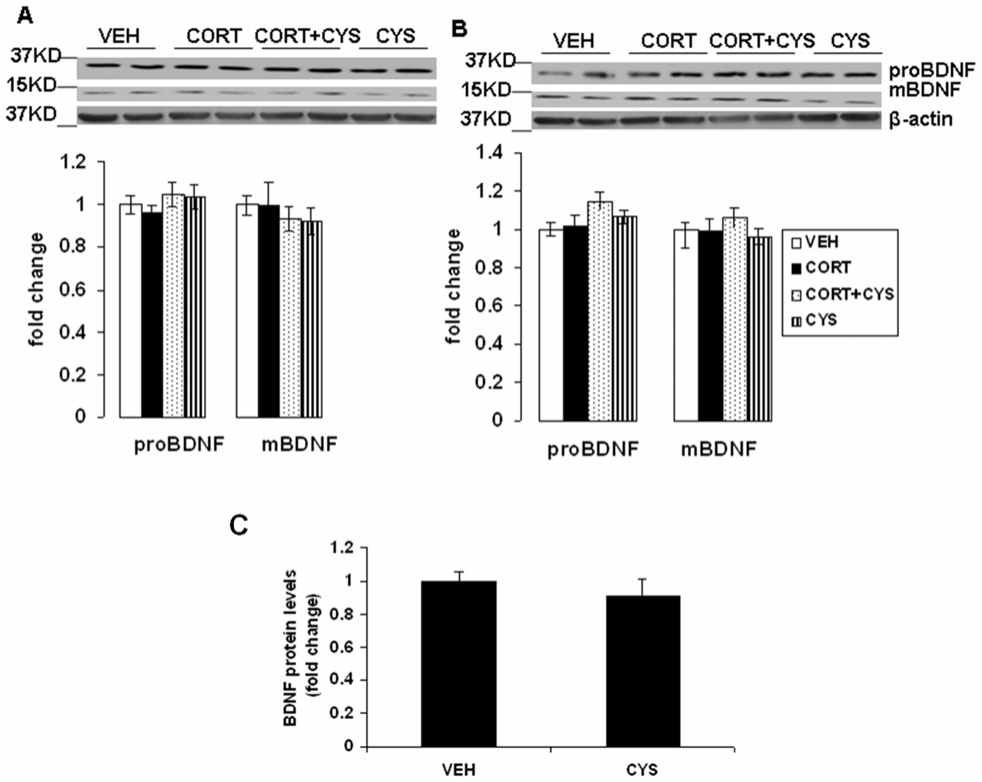
Effects of cysteamine in chronic corticosterone-treated mice on proBDNF and mature BDNF (mBDNF) protein levels in the frontal cortex and hippocampus. CD-1 male mice were treated for 7 weeks with vehicle (0.45% hydroxypropyl-β-cyclodextrin) or corticosterone (CORT; 35 ug/ml) in the presence or absence of cysteamine (CYS; 150 mg/kg/day) during the last three weeks of corticosterone treatment. proBDNF and mBDNF protein levels were determined in the (A) frontal cortex and (B) hippocampus by Western blot analysis. The upper panels shows a representative autoradiogram of proBDNF and mBDNF and the lower panel represents the fold change in optical density values normalized to vehicle-treated controls. β-actin was used as a protein loading control. Values are mean ± SE (n = 6 mice per group). (C) BDNF protein levels as measured by ELISA in frontal cortex samples from mice treated with vehicle or cysteamine for 3 weeks as above. Data represent the fold change in BDNF protein levels (pg/mg protein) normalized to vehicle-treated controls. Values are mean ± SE (n = 5 mice per group).

We also determined whether treatment with corticosterone and/or cysteamine in mice alters BDNF levels in frontal cortex and hippocampus. We measured BDNF protein levels using western blots instead of routine ELISA methods to determine the difference in proBDNF and mature BDNF protein levels. Two-way ANOVA on proBDNF levels in the frontal cortex did not find any significant main effect of pretreatment [F(1,18) = 0.0604; p = 0.808], treatment [F(1,18) = 1.4; p = 0.253] and pretreatment x treatment interaction [F(1,18) = 0.219; p = 0.645]. Similarly, the data on mature BDNF levels in frontal cortex did not yield significant main effect of pretreatment [F(1,18) = 0.0062; p = 0.938], treatment [F(1,18) = 1.05; p = 0.318] and pretreatment x treatment interaction [F(1,18) = 0.0165; p = 0.899]. Two-way ANOVA revealed a significant main effect of treatment [F(1,18) = 5.77; p<0.01] on proBDNF levels in hippocampus, but no effect of pretreatment [F(1,18) = 1.709; p = 0.208] and pretreatment x treatment interaction [F(1,18) = 0.348; p = 0.562]. We did not find any significant main effect of pretreatment [F(1,18) = 0.513; p = 0.483], treatment [F(1,18) = 0.0645; p = 0.802] and pretreatment x treatment interaction [F(1,18) = 0.696; p = 0.415] on BDNF levels in hippocampus. Post-hoc analysis did not show any significant change in proBDNF and mature BDNF protein levels in corticosterone or corticosterone plus cysteamine treated mice as compared to vehicle-treated mice in frontal cortex and hippocampus (Fig 6). Since we did not find any change in BDNF protein levels in western blot analysis, we further examined the BDNF levels by ELISA using frontal cortex tissue samples from mice treated with vehicle or cysteamine for 3 weeks. We did not find any significant change in BDNF levels between vehicle and cysteamine treated groups (Fig 6C). We also examined the effects of cysteamine on plasma corticosterone levels. As we reported earlier [16], we found a significant decrease in plasma corticosterone levels following corticosterone treatment. However, cysteamine had no effect on plasma corticosterone levels in vehicle-treated and corticosterone-treated mice (data not shown).

### Cysteamine reverses the chronic corticosterone-induced anxiety/depression-like phenotype in mice

A number of studies have shown that chronic glucocorticoid treatment induces anxiety/depression-like behaviors in animals [2], [17]. In the present study, we investigated the effect of cysteamine on corticosterone treatment-induced anxiety-like behaviors in an open field (OF), Light/Dark test and Elevated Plus maze. To determine the effect of cysteamine on depression-related behavior, mice were tested in the tail suspension test (TST), a well-established paradigm in which reductions in immobility reflect antidepressant-like activity.

**Figure 7:**
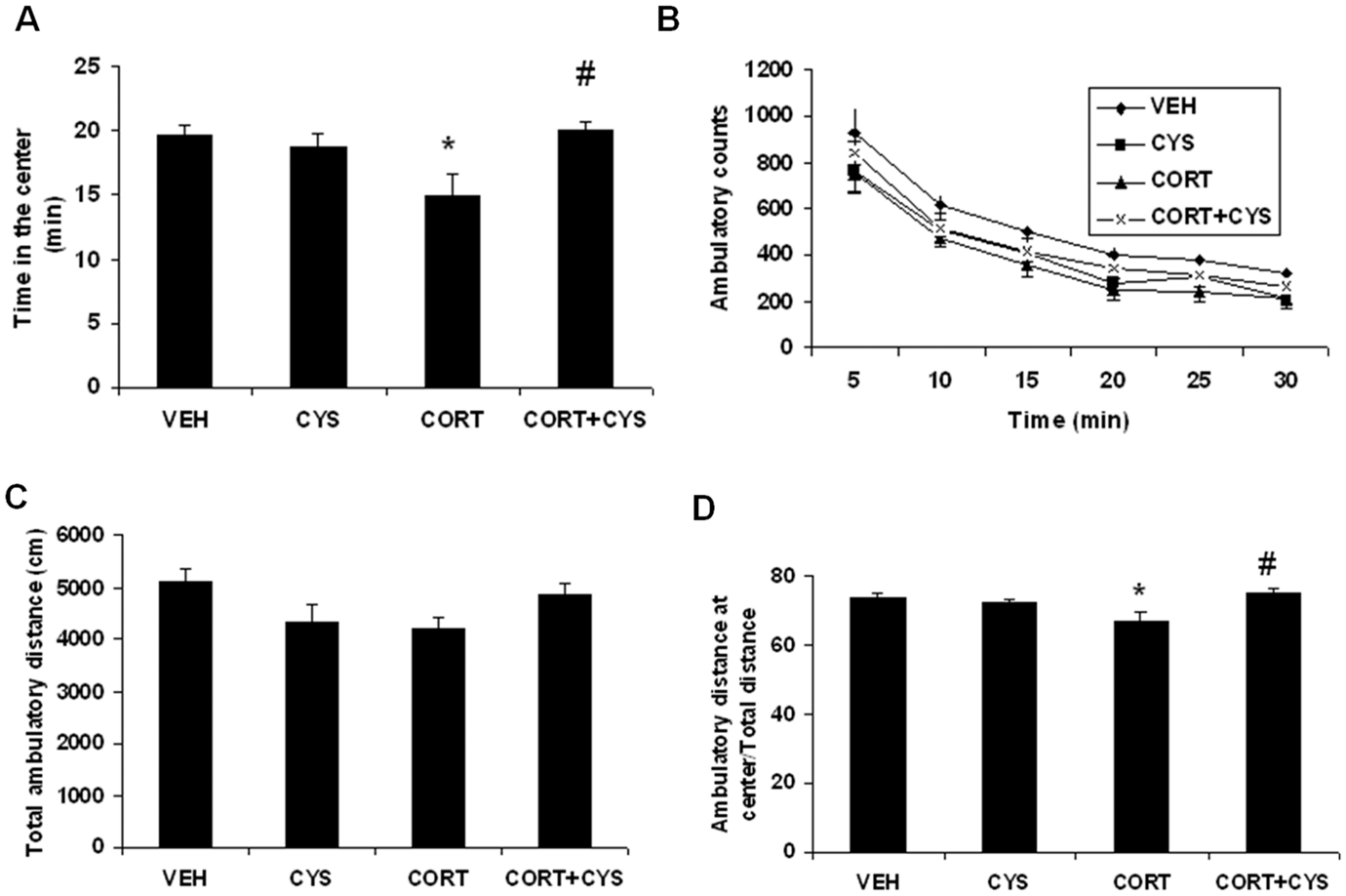
Effects of cysteamine in chronic corticosterone-treated mice in the Open Field test. CD-1 male mice were treated for 7 weeks with vehicle (0.45% hydroxypropyl-β-cyclodextrin) or corticosterone (CORT; 35 ug/ml) in the presence or absence of cysteamine (CYS; 150 mg/kg/day) during the last three weeks of the corticosterone treatment. (A) Mean total of the time-spent in the center for the entire session, (B) the ambulatory counts for each 5 min period, (C) the total ambulatory distance and (D) the ambulatory distance in the center over total. Values plotted are mean ± SEM (n = 8–10 per group). Bonferroni's post hoc test. *p<0.05 versus vehicle and ^#^p<0.05 versus CORT.

Fig 7 illustrates the effects of drug treatment on open field locomotor activity. Mice treated with corticosterone for 7 weeks showed significant changes in open field activity behavior. Statistical analysis of the data on time spent in center by two-way ANOVA with corticosterone/vehicle pretreatment and cysteamine/vehicle as main effects revealed a significant main effect of treatment [F(1,30) = 5.675; p<0.01] and pretreatment x treatment interaction [F(1,30) = 6.72; p<0.01], but no significant effect of pretreatment [F(1,30) = 2.198; p = 0.148]**.** Subsequent Post hoc analysis showed that corticosterone-treated mice spent less time in the center as compared to vehicle-treated mice (Fig 7A; p<0.05). Interestingly, cysteamine treatment reversed this anxiety-related behavior (p<0.01 verses corticosterone group in time spent in the center). There was clear evidence of habituation to the open field environment in all test groups as indicated by the diminishing horizontal counts over time (Fig 7B). Two-way ANOVA revealed a significant main effect of treatment [F(3,177) = 9.166; p<0.0001] and time [F(5,177) = 61.49; p<0.0001] without a significant treatment x time interaction [F(15,177) = 0.111; p = 1.0]. However, post hoc analysis did not show any significant change in ambulatory counts between treatment groups. In addition, we did not find any significant change in total ambulatory distance between treatment groups (Fig 7C). Next, we examined the ratio of ambulatory distance in center to total distance. Two-way ANOVA showed a significant main effect of treatment [F(1,30) = 4.512; p<0.01] and pretreatment x treatment interaction [F(1,30) = 9.481; p<0.001], but no significant effect on pretreatment [F(1,30) = 1.878; p = 0.182]. Post-hoc analysis Corticosterone-treated mice showed a decrease in the ratio (Fig 7D; p<0.05) and cysteamine treatment reversed this behavior (p<0.05). These data suggest that cysteamine treatment reverses chronic corticosterone-induced changes in open field activity behavior in mice.

**Figure 8:**
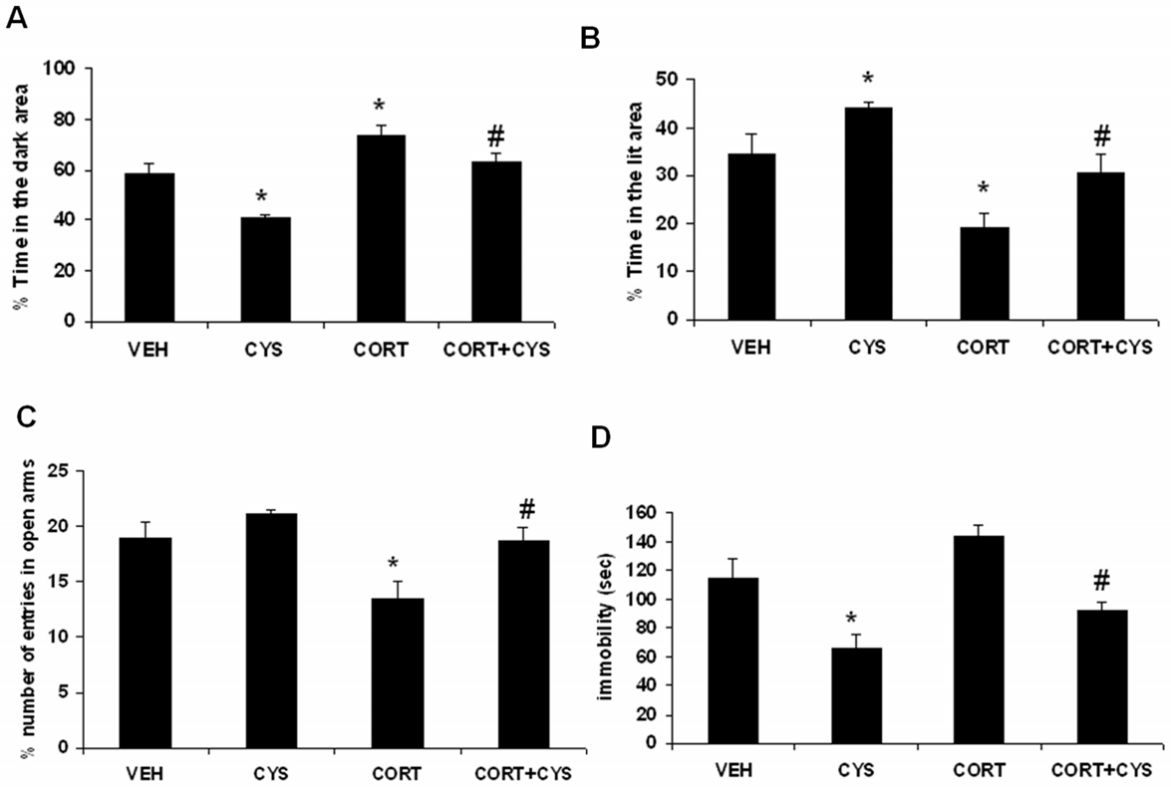
Effects of cysteamine in chronic corticosterone-treated mice on anxiety-like behaviors as measured in the (A-B) Light/Dark test, (C) Elevated Plus Maze Maze test and (D) Tail Suspension Test. CD-1 male mice were treated for 7 weeks with vehicle (0.45% hydroxypropyl-β-cyclodextrin) or corticosterone (CORT; 35 ug/ml) in the presence or absence of cysteamine (CYS; 150 mg/kg/day) during the last three weeks of the corticosterone treatment. (A) % of the time spent in the dark area, (B) % of the time spent in the lit area; (B) % of the number of entries in the open arms, and (D) the immobility score (in seconds). Values are mean ± SE (n = 8–9 mice per group). Bonferroni's post hoc test. *p<0.05 versus vehicle and ^#^p<0.05 versus CORT.

In Light/Dark Box Assessment, statistical analysis by two-way ANOVA provided the following results in the time spent in the brightly lit area, main effect of pretreatment, [F(1,30) = 20.97; p<0.0001]; treatment[F(1,30) = 19.25; p<0.0001], and pretreatment x treatment interaction, [F(1,30) = 0.0359; p = 0.851]]. Data on the time spent in the dark area showed a significant main effect of pretreatment [F(1,30) = 34.53; p<0.0001] and treatment [F(1,30) = 21.32; p<0.0001], but no significant effect of pretreatment x treatment interaction [F(1,30) = 1.143; p = 0.2943]. Post hoc analyses indicated that corticosterone treated mice spent significantly more time in the dark (Fig 8A; p<0.05) and less time in the brightly lit zone (Fig 8B; p<0.05) when compared to vehicle controls. Further, this increased preference for the dark zone was reversed by cysteamine treatment (p<0.05), again indicating that cysteamine attenuated the pro-anxiety effects of corticosterone.

To further validate the potential of cysteamine in reversing the anxiety-like behavior induced by chronic corticosterone treatment, we next tested the effects of cysteamine in chronic corticosterone treated mice in an elevated plus maze test. Two-way ANOVA provided the following results in the number of entries into the open arms, main effect of pretreatment [F(1,30) = 12.65; p<0.01]; treatment [F(1,30) = 8.964; p<0.01], and pretreatment x treatment interaction [F(1,30) = 1.185; p = 0.286]. We found that cysteamine reversed the corticosterone-induced reductions in the number of entries into the open arms (Fig 8C;p<0.05).

Two-way ANOVA on TST showed a a significant main effect of pretreatment [F(1,24) = 6.838; p<0.01] and treatment [F(1,24) = 22.25; p<0.001], but no significant effect on pretreatment x treatment interaction [F(1,24) = 00026; p = 0.960]. Post-hoc analyses revealed that chronic corticosterone for 7 weeks did not result any significant change in immobility score in TST (Fig 8D). However, cysteamine treatment decreased the duration of immobility in both vehicle (p<0.05) as well as corticosterone treated mice (p<0.05).

### TrkB is necessary for anxiolytic/antidepressant effects of cysteamine

**Figure 9:**
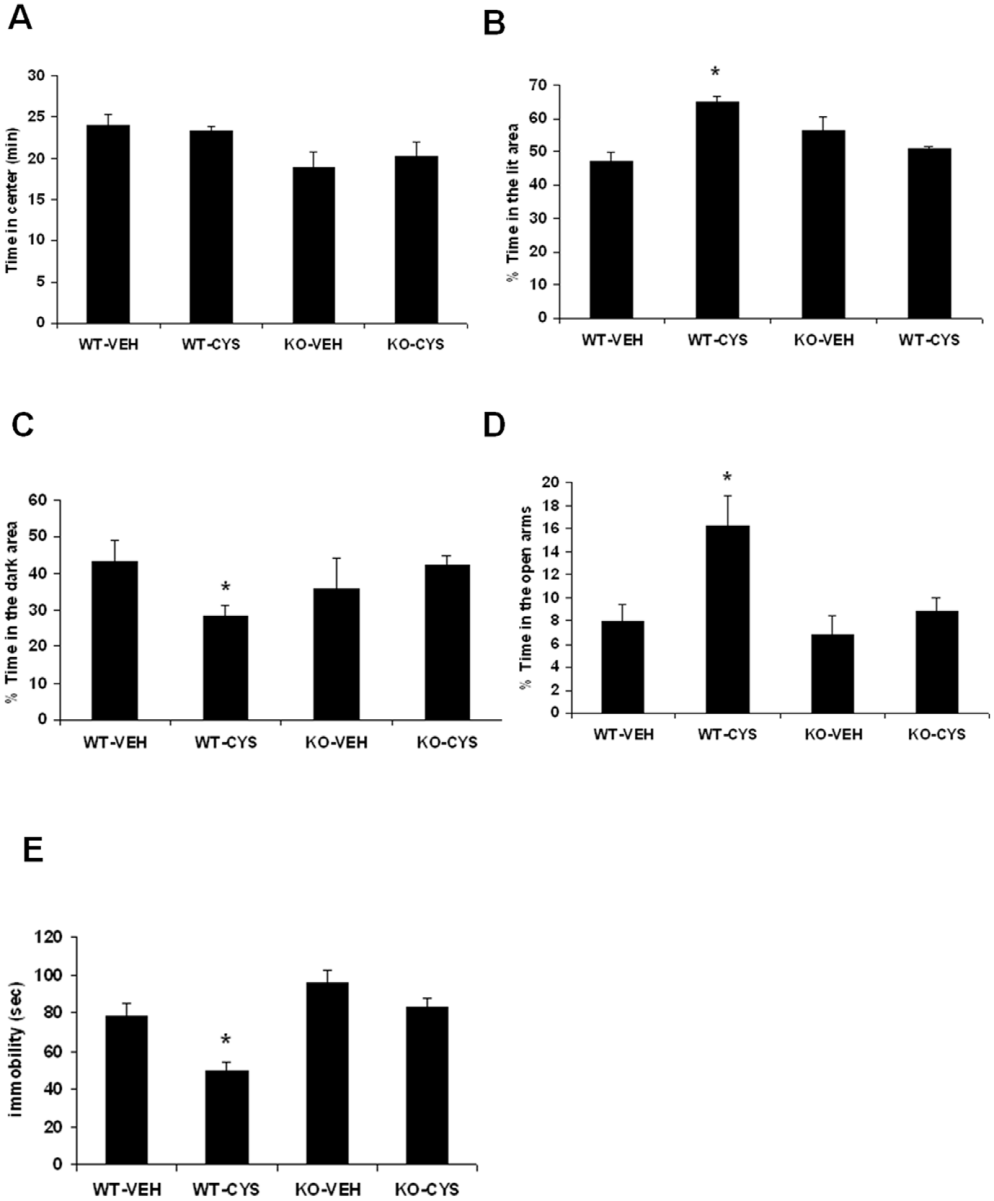
Effects of cysteamine in TrkB knockout mice on anxiety-like behaviors as measured in the (A) Open Field test, (B-C) Light/Dark test, (D) Elevated Plus Maze Maze test and (E) Tail Suspension Test. TrkB knock out (KO) and wild type (WT) male mice were treated for 3 weeks with vehicle (water) or cysteamine (CYS; 150 mg/kg/day). (A) Mean total of the time spent in the center for the entire session, (B) % of the time-spent in the lit area, (C) % of the time-spent in the dark area, (D) % of the time in open arms, and (E) the immobility score (in seconds). Values are mean ± SE (n = 6 mice per group). Bonferroni's post hoc test. *p<0.05 versus vehicle.

We next examined the role of TrkB in mediating the behavioral effects of cysteamine treatment in mice. In OF, two-way ANOVA with genotype and treatment as the main effects showed no significant main effect of genotype [F(1,18) = 1.329; p = 0.264], treatment [F(1,18) = 0.464; p = 0.504] and genotype x interaction [F(1,18) = 0.0312; p = 0.861]. Post-hoc analysis did not find any significant difference in the time spent in the center between vehicle-treated WT and TrkB KO mice (Fig 9A). The lack of change in open field activity was further tested by examining the ratio of ambulatory distance in center to total distance. TrkB knockout mice did not exhibit any significant change in the above phenotype (data not shown). In addition, we did not find any significant change in the above measures following cysteamine treatment indicating that TrkB knockout mice are not responsive to cysteamine in open field activity.

Since C57BL/6 mice (the strain background for TrkB knockout mice) are known to respond poor in open filed activity to antidepressant treatments [18], we next tested the anxiety phenotype of TrkB knockout mice using two additional behavioral measures: light/dark test and elevated plus maze. In the light/dark test, two-way ANOVA provided the following results in the time spent in brightly lit area, main effect of genotype, [F(1,18) = 0.879; p = 0.361], treatment, [F(1,18 = 5.560; p<0.01]; genotype x treatment interaction, [F(1,18) = 20.45; p<0.001]. Data on the time spent in the dark area showed a significant effect of genotype x treatment interaction [F(1,18) = 22.54; p<0.001], but no significant effect of genotype [F(1,18) = 2.1; p = 0.164] and treatment [F(1,18) = 3.359; p = 0.076]. Post hoc analyses indicated that vehicle treated TrkB knockout mice do not display an anxious-like phenotype as assessed by the time spent in light and dark zones (Fig 9B-C). However, we found a significant increase in time spent in the brightly lit area in control mice following cysteamine treatment (Fig 9B; p<0.01) that was absent in the TrkB knockout mice. Similarly, control mice treated with cysteamine spent less time in the dark zone compared to vehicle treated control mice (Fig 9C; p<0.01). We next tested the effect of cysteamine in TrkB knockout mice in an elevated plus maze. Two-way ANOVA showed significant main effect of genotype [F(1,18) = 7.836; p<0.01] and treatment [F(1,18) = 0.5.412; p<0.01] , but no significant effect of genotype x interaction [F(1,18) = 2.793; p = 0.120]. Similar to open field and light/dark tests, we did not find any significant difference in the elevated plus maze test between wild-type and TrkB knockout mice (Fig 9D). Furthermore, while in wild-type mice cysteamine significantly increased the % of time spent in the open arms (p<0.05), cysteamine had no effect in mutant mice. Taken together, these data indicate that TrkB is required for the behavioral effects of cysteamine in the Light/Dark and EPM.

The role of TrkB in the behavioral effects of cysteamine was further examined by assessing behavior in TST. Two-way ANOVA showed significant main effect of genotype [F(1,18) = 20.27; p<0.001] and treatment [F(1,18) = 13.85; p<0.01] , but no significant effect of genotype x interaction [F(1,18) = 2.112; p = 0.165]. TrkB knockout mice showed a nonsignificant increase in immobility relative to wild type mice (Fig 9E; p = 0.09). Moreover, cysteamine significantly decreased the immobility in control littermates (p<0.05), but TrkB knockout mice did not respond to cysteamine.

## Discussion

The data from our study provide evidence for the potential of cysteamine to attenuate corticosterone-induced changes in TrkB levels as well as anxiety and depression-related behaviors in mice. Our data also suggest that mice lacking TrkB show reduced responses to cysteamine treatment in behavior tests.

Our observations of an anxiety-like phenotype in mice following corticosterone exposure are in agreement with previous findings [2], [17]. A number of studies have suggested that corticosteroids are involved in the anxiety-like features that are frequently comorbid with psychiatric disorders such as depression [19]. In addition, studies have shown that chronic corticosteroid exposure, but not acute exposure manifests an anxiety-like phenotype in rodents [2], [17]. Our recent study indicated a decrease in serum corticosterone levels in mice following corticosterone exposure for 7 weeks [16]. This could be due to the fact that exogenous corticosterone treatment can flatten the diurnal corticosterone rhythm rather than result in an absolute increase in circulating corticosterone levels [20]. Moreover, corticosterone administration has been shown to inhibit the normal p.m. rise in peripheral corticosterone levels in rodents (animals were sacrificed between 3 and 4 pm in our study), whereas control animals demonstrate a normal circadian rhythm of corticosterone [21]. Consistent with previous report [2], our study indicates that chronic corticosterone treatment in mice does not result any significant change in immobility score in TST.

Using a chronic corticosterone-exposed mouse model, we provide evidence for anxiolytic-like potential of cysteamine, in OF, Light/dark and EPM tests. It is interesting to note that vehicle-treated mice were found nonresponsive to cysteamine in OF and EPM tests. However, when pretreated with corticosterone for 4 weeks, these mice responded well to cysteamine suggesting the involvement of two different mechanistic pathways in cysteamine's action in pathological vs normal conditions. Although chronic corticosterone exposure was not associated with significant change in the immobility score in the TST, cysteamine was effective in reducing the immobility score in both corticosterone-treated and vehicle-treated mice.

TrkB plays an important role in stress-induced changes in neuroplasticity [22], 2010) and TrkB alterations have been linked to a number of stress related psychiatric disorders [23]. The expression of full-length TrkB was significantly lower in the PFC and hippocampus of adult suicide subjects compared with age-matched healthy controls [24]. To determine whether chronic corticosterone exposure induces changes in TrkB expression, we analyzed TrkB mRNA and protein levels in the frontal cortex and hippocampus, two brains structures involved in the stress response [25]. We did not find any significant change in TrkB mRNA levels following corticosterone treatment. This finding suggests that chronic corticosterone affects the rate of TrkB protein synthesis or degradation. Although the mechanism of corticosterone-induced TrkB protein downregulation is unknown, the role of ubiquitin-proteasome pathway has been suggested for ligand-induced TrkB degradation [26]. Further studies are necessary to determine whether ubiquitin system is involved in chronic corticosterone-induced TrkB regulation. We found a significant reduction in TrkB protein levels both in frontal cortex and hippocampus following chronic corticosterone treatment. Interestingly, TrkB knockout mice showed a reduced response to cysteamine in the light/dark, EPM and TST paradigms. This suggests that TrkB might have a role in mediating anxiolytic/antidepressant actions of cysteamine.

Recent studies indicate that cysteamine increases brain BDNF levels in rodents [14]-[15]. Moreover, acute cysteamine treatment has been shown to increase hippocampal BDNF levels and reduce immobility time in FST and TST in mice [27]. However, our study failed to find any significant difference in BDNF levels in frontal cortex and hippocampus following cysteamine treatment. Earlier studies have determined BDNF levels by routine ELISA methods, which are poor in differentiating pro and mature BDNF levels. To test the above possibility, we carried out BDNF analysis by both immunoblot and ELISA methods. However, we could not find any significant differences in BDNF levels between vehicle-treated and cysteamine-treated mice. The other possible factor for the observed discrepancy in BDNF levels between our results and those of other studies could be the duration of cysteamine treatment. The duration in our study was 3 weeks whereas the duration in the other two studies was 7 days. In addition, the time of animal sacrifice has been shown to play an important role in the regulation of BDNF expression. BDNF mRNA levels in animals chronically treated with duloxetine and killed 1 h after the last administration of the antidepressant were significantly higher than in those killed at 24 h [28].

In conclusion, the results of this mouse study indicate a potential for cysteamine to effectively treat corticosterone-related anxiety and depression symptoms. Our study indicates that cysteamine treatment improved the anxiety/depression behavior in both control and corticosteroid-treated animals. The overall hypothesis of the study was to find whether cysteamine could reverse stress-induced changes in anxiety/depression-related behavior. As a first step in this line of research, we have selected corticosterone-induced mice as a model to test our hypothesis. We need to perform additional studies using other stress models relevant to anxiety/depression to validate the potential of cysteamine in stress-related psychiatric disorders. In addition, it would also be interesting to determine whether cysteamine could reverse other corticosterone-induced behaviors that are related to depression or schizophrenia. Based on the data we have collected to date, it would be premature to make the statement that cysteamine will be more effective than the currently available antidepressants. However, all of the available antidepressant drugs (i.e., both the newer and older generation agents) have adverse effects (e.g., sedation, weight gain, sexual side effects) as well as other limitations (e.g., drug interactions). Cysteamine might represent a new class of antidepressants that is not structurally related to any of those currently marketed. Therefore it may be of interest to further evaluate this compound both for antidepressant and anxiolytic properties. Since cysteamine tolerability has previously been demonstrated in human subjects (e.g., Huntington Disease patients), the animal studies described here highlight the potential use of this compound as a novel therapeutic strategy for glucocorticoid-related symptoms of psychiatric disorders.

## Methods

### Ethics Statement

All experiments in the present study were conducted with the approval of the Medical College of Georgia, Committee on Animal Use for Research and Veterans Affairs Medical Center Subcommittee on Animal Use (Protocol # BR10-11-377 dtd. 11/17/2010).

### Animals

CD-1 male mice (25–30 g) were purchased from Charles River Laboratories, Wilmington, MA, USA. TrkB knockout (TrkB^-/-^) mice were provided by Dr. Barbara Rohrer, Medical University of South Carolina, Charleston, SC and the colony was maintained in our animal housing facility at the Georgia Health Sciences University. The generation of mice lacking the TrkB has been described previously [29]. Male wild-type (WT) and TrkB^-/-^ mice (C57BL/6 strain background) used in a given experiment, originated from the same breeding series and were matched for age and weight (age = 2–3 months; weight = 25–30 g). Animals were housed 4 mice per cage with water and food available *ad libitum*. Mice were maintained on a 12-h light–dark cycle with the lights on at 0700 hours. All experimental procedures were performed during the light cycle.

### Drug treatment

Fig 1 describes the experimental design used for the drug treatment. Corticosterone (Sigma, St Louis, MO) was dissolved in 0.45% hydroxypropyl-β-cyclodextrin (Sigma), where as cysteamine (Sigma) was dissolved in water. In corticosterone treatment studies, CD-1 mice were treated for 7 weeks with vehicle or corticosterone (35 ug/ml equivalent to 5 mg/kg/day) in the presence or absence of cysteamine (150 mg/kg/day) during the last three weeks of the corticosterone treatment. In separate set of experiments, WT as well as TrkB KO mice were administrated with vehicle or cysteamine (150 mg/kg/day) for 3 weeks. The vehicle as well as drug solutions were delivered *ad libitum* in the drinking water. The dose and duration of corticosterone treatment was selected based on an earlier study [2] where the above dose and duration of treatment with corticosterone induced anxiety and depression-like behaviors in mice. The cysteamine dose was selected based on earlier studies where this concentration was found to be non-toxic, but showed neuroprotective effects [14]–[15], [30]. All animals were monitored for change in body weight and food intake daily as possible adverse effects of the treatment, and adjustments were made in the amount of drug depending upon the fluid consumed and weight of the animals. At the end of the treatment, the same animal was successively tested in the Open Field (OF) paradigm, the Light/dark test, the Elevated Plus Maze test (EPM), the Tail Suspension Test (TST) and then sacrificed for protein or mRNA analysis.

### Western blot analysis

Animals were sacrificed by cervical dislocation, and frontal cortex and hippocampus samples from vehicle as well as drug-treated mice were collected according to a mouse brain atlas [31]. The tissue samples were homogenized in ice-cold buffer (10 mM Tris–HCl, pH 7.5, 150 mM NaCl, 0.1% SDS, 1% Nonidet P-40, 1% sodium deoxycholate) supplemented with protease inhibitor cocktail (Sigma) containing 104 mM AEBSF, 0.08 mM aprotinin, 2 mM leupeptin, 4 mM bestatin, 1.5 mM pepstatin A, and 1.4 mM E-64. After a 15-min incubation on ice, the extracts were clarified by centrifugation at 14,000 rpm for 15 min at 4 °C and stored at −70°C. Protein concentrations were determined by the bicinchoninic acid method (BCA Protein Assay Kit, Sigma, USA). Equal amounts of protein were resolved in SDS-polyacrylamide gels and transferred electrophoretically onto a nitrocellulose membrane (Bio-Rad). Membranes were blocked for 1 h in TBST (10 mM Tris–HCl, pH 8.0, 138 mM NaCl, 2.7 mM KCl, and 0.05% Tween-20) and 5% non-fat milk and incubated overnight with the indicated antibodies. The primary antibodies used were anti-TrkB (1∶400; #4603; Cell Signaling, MA, USA), anti-BDNF (1∶250; sc-546; Santa Cruz Biotech), or anti-β-actin (1∶1500; A-5441; Sigma). After washing with TBST, the membranes were incubated for 1 h with horseradish peroxidase-conjugated anti-rabbit or anti-mouse anti-sera in TBST and 3% non-fat milk. The membranes were washed again with TBST, and proteins were visualized by enhanced chemiluminescence. The densitometric values for the proteins of interest were corrected for protein loading using β-actin. TrkB antibody shows two bands in the western blots, one at ∼98 KDa for truncated TrkB and the other at ∼145 KDa for full-length TrkB receptor. We have selected the full-length receptor band for our analyses.

### RNA isolation and qRT-PCR

Total RNA was isolated from frontal cortex and hippocampal areas using a commercially available kit (SV RNA Isolation, Promega, Madison, WI), according to the manufacturer's instructions. qRT-PCR was performed on a Mastercycler® ep realplex ^2^ S (Eppendorf, Cepheid, Sunnyvale, CA) using a SuperScript III Platinum SYBR Green One-Step qRT-PCR kit (Invitrogen, Carlsbad, CA). Primers utilized were as follows: TrkB FL (2257–2622 bp, Gen Bank accession No: NM_001025074, forward primer 5′-GACAATGCACGCAAGGACTT-3′; reverse primer 5′-AGTAGTCGGTGCTGTACACA-3′) and housekeeping gene, ribosomal protein S3 (RPS3) (284–466 bp, Gen Bank accession No: NM_012052, forward primer 5′-AATGAACCGAAGCACACCATA-3′; reverse primer 5′-ATCAGAGAGTTGACCGCAGTT-3′). Samples were quantified using serial cDNA dilutions, and data were normalized to RPS3, which was unaffected by experimental manipulations [32]. Data were expressed as fold change versus WT mice. Primer specificity was confirmed by melting curve analysis and electrophoresis of PCR products on a 2% agarose gel to confirm the presence of a single band of the predicted size.

### Brain-Derived Neurotrophic Factor Immunoassay

Brain-derived neurotrophic factor protein was measured with a conventional sandwich ELISA using the BDNF Emax ImmunoAssay System (Promega, Madison, WI, USA) according to the protocol of the manufacturer.

### Behavioral Experiments

All behavioral experiments were conducted in rooms equipped with white noise generators (San Diego Instruments, San Diego, CA) set to provide a constant background level of 70 dB and ambient lighting of approximately 25–30 Lux (lumen/m^2^). Test subjects were handled daily for several minutes (each) for at least one week prior to experimentation. Animals were transferred (in their home cages) to the behavioral testing rooms each morning approximately 30 min before the beginning of experiments. The behavioral tests were performed in the following order: light/dark test, elevated plus maze test, Open field test and tail suspension test. The animals were given two days to recover before the next behavioral test. All procedures employed during this study were reviewed and approved by the Medical College of Georgia Institutional Animal Care and Use Committee and are consistent with AAALAC guidelines. Measures were taken to minimize pain or discomfort in accordance with the National Institute of Health Guide for the Care and Use of Laboratory Animals (NIH Publications No. 80-23) revised 1996. Significant efforts were also made to minimize the total number of animals used while maintaining statistically valid group numbers.

#### Open Field Activity

Mouse open field activity monitors (27.9 cm x 27.9 cm, Med Associates St Albans, VT) were used for these experiments. The following parameters were recorded for the 30 min test session: horizontal activity (horizontal photobeam breaks or counts) and vertical activity (vertical photobeam breaks). Thus, spontaneous locomotor activity, olfactory activity (rearing and sniffing movements) were assessed. The time spent in the central and peripheral zones of the apparatus (defined areas represented approximately 75% and 25% of the total floor area, respectively) was also recorded as an anxiety-related behavioral assessment. The first exposure of an animal to the open field can be used to compare group-related differences in emotionality induced by exposure to a novel environment. Thigmotaxis (i.e., the tendency of rodents to stay in close contact with the walls of the open field in which they are placed is thought to reflect their underlying propensity to avoid open, unknown and potentially dangerous areas [33].

#### Light-Dark Preference Test

To further assess the effects of drug treatment on anxiety levels, a Light/Dark Preference Test (also referred to as light/dark exploration or emergence neophobia test) was conducted. This test is one of the most commonly-used rodent models of anxiety [34]–[35]; and avoidance of the lighted portion of the chamber reflects elevated anxiety while significantly reduced time spent in the dark area reflects an anti-anxiety effect of a test drug. In these experiments, the mouse open field activity monitors described above were fitted with dark box inserts (which are opaque to visible light) to cover one-half of the open field area thus separating the apparatus into two zones of equal area (i.e., a brightly lit zone and a darkened zone). Desk lamps located above the activity monitors were used to provide an illumination level of approximately 1000 lux in the brightly lit zone, whereas the illumination level in the darkened zone was approximately 5 lux. The time spent and distance traveled in the light and dark zones of the apparatus were recorded for the 10 min test session.

#### Elevated Plus Maze

The elevated plus maze is another widely used anxiety paradigm based on unconditioned responses of rodents to a potentially dangerous environment. The maze exploits the fear of open, and exposed areas (as described above) as well as the fear of heights [36]. The plus maze comprises two (opposite) closed and two open arms, each arm located on a central pole. The combination of height, luminosity and open space is assumed to induce fear or anxiety in the rodent. The degree of anxiety is thus assessed by measuring the time spent on the open and closed arms and the number of entries made into each arm. The elevated plus maze consisted of two orthogonal closed (15 cm×6 cm×30 cm) and open (1 cm×6 cm×30 cm) arms forming a cross, with a quadrangular (6 cm×6 cm) area located at the intersection. The maze was placed 50 cm above the floor and was made of black plastic. Each test lasted 10 min, and was initiated by placing a mouse on the center facing an open arm. The behavior of the animal was recorded with a video camera and later scored by an independent observer blind to treatment. The following behaviors were scored and compared between groups: open and closed arm entries (defined as all 4 paws passing the threshold between the center/intersection portion of the maze and the beginning a particular arm), time spent and distance traveled in the closed and open arms as well as in the center area.

#### Tail suspension test

Animals were tested using the tail suspension test to assess depressive-like behavior. Each mouse was suspended by its tail from an aluminum bar and secured with adhesive tape. Behavior was recorded for 6 min. Observers blinded to the subject's treatment group scored video recordings for time spent struggling or immobile.

### Statistical Analyses

Data are expressed as mean±SE. For all experiments one-way or two-way ANOVA with repeated measure were applied to the data as appropriate with Bonferroni's multiple comparison test for post hoc analysis. Individual comparisons between two groups were performed with Student'st test. Probability (*P*) values of less than 5% were considered significant.
